# PHLDA1-PRDM1 mediates the effect of lentiviral vectors on fate-determination of human retinal progenitor cells

**DOI:** 10.1007/s00018-024-05279-z

**Published:** 2024-07-16

**Authors:** Xing Hu, Jia Chen, Wangxuan Dai, Yuhua Xiao, Xu Chen, Zheyao Chen, Shuyao Zhang, Youjin Hu

**Affiliations:** 1 State Key Laboratory of Ophthalmology, Zhongshan Ophthalmic Center, Sun Yat-Sen University, Guangzhou, China; 2 Guangdong Provincial Key Laboratory of Ophthalmology and Visual Science, Guangzhou, China

**Keywords:** Lentiviral vectors, PHLDA1, Photoreceptor specification, Single-cell transcriptomic sequencing, Human retinal organoid

## Abstract

Lentiviral vectors have markedly enhanced gene therapy efficiency in treating congenital diseases, but their long-term safety remains controversial. Most gene therapies for congenital eye diseases need to be carried out at early ages, yet the assessment of related risks to ocular development posed by lentiviral vectors is challenging. Utilizing single-cell transcriptomic profiling on human retinal organoids, this study explored the impact of lentiviral vectors on the retinal development and found that lentiviral vectors can cause retinal precursor cells to shift toward photoreceptor fate through the up-regulation of key fate-determining genes such as *PRDM1*. Further investigation demonstrated that the intron and intergenic region of *PRDM1* was bound by *PHLDA1*, which was also up-regulated by lentiviral vectors exposure. Importantly, knockdown of *PHLDA1* successfully suppressed the lentivirus-induced differentiation bias of photoreceptor cells. The findings also suggest that while lentiviral vectors may disrupt the fate determination of retinal precursor cells, posing risks in early-stage retinal gene therapy, these risks could potentially be reduced by inhibiting the PHLDA1-PRDM1 axis.

**Supplementary Information:**

The online version contains supplementary material available at 10.1007/s00018-024-05279-z.

## Introduction

Gene therapy presents promising treatment options for numerous severe diseases by introducing normal protein-coding genes to treat hereditary diseases. Notably, this therapeutic approach has demonstrated improved retinal function in patients with Leber’s congenital amaurosis (LCA) [1, 2] and has shown anti-leakage activity in neovascular age-related macular degeneration [3].Currently, widely used viral vectors in gene therapy include adenovirus, lentivirus, and adeno-associated virus. Among these, lentivirus, an improved retrovirus derived from HIV, stands out for its effective gene transfer, stable expression [4], and relatively large transgene carrying capacity [5]. Nevertheless, previous studies have raised concerns about lentiviral vector-mediated transduction, highlighting possible adverse reactions such as tumor formation [6], immune response, retinal degeneration [7], and the exacerbation of retinal pigment epithelial atrophy [8]. Importantly, gene therapies for genetic developmental disorders, such as enhanced S-cone syndrome caused by *NRL* mutations [9] and photoreceptor ciliary defects caused by *CEP290* mutations [10], need to be carried out at early ages. However, the assessment of related risks to ocular development posed by lentiviral vectors is challenging. A comprehensive understanding of the impact of lentiviral infection on the human developing retina is important for a thorough assessment of gene therapy in the retinal genetic diseases.

Due to ethical, technical, and biological limitations, it is not feasible to assess the impact of lentiviral infection on the developing system in vivo. While animal models such as mice have been widely used for in vivo experiments, significant differences in molecular characteristics and structure of rodent and human retinas [11] pose challenges in fully evaluating the impact of lentiviral vectors on retinal development. The development of human retinal organoid culture technology has provided a potential solution, as human retinal organoids can resemble the development of the retina and possess mature photoreceptor cells [12]. Compared to mice, retinal organoids have a more similar developmental sequence, cell types, and layered structure, providing an ideal model to replicate the development process of human embryonic retina in vitro [13–15]. Moreover, the lack of immune cells in the development of human retinal organoids eliminates the confounding effects of immune responses induced by lentivirus infections. This permits a clearer assessment of the lentiviral vectors’ influence on retinal neuron development without the variability introduced by immune-mediated reactions [16].

In this study, we used human retinal organoids as a model to evaluate the effects of lentiviral vectors on retinal development. Our results demonstrated that lentiviral infection can disturb the differentiation of retinal progenitor cells (RPCs) into photoreceptor cells. Single-cell transcriptome analysis revealed that lentiviral vectors can stimulate the up-regulation of the key gene *PHLDA1*, inducing the activation of gene transcription networks associated with photoreceptor cell specialization. Furthermore, by binding to the intron and intergenic regions of *PRDM1*, PHLDA1 can upregulate PRDM1 expression, thereby regulating the fate of photoreceptor cell specialization. The findings also suggest that while lentiviral vectors may disrupt the fate determination of retinal precursor cells, posing risks in early-stage retinal gene therapy, these risks could potentially be reduced by inhibiting the PHLDA1-PRDM1 axis.

## Materials and methods

### Cell culture

Y79 cells were cultured in RPMI 1640 medium supplemented with 15% fetal bovine serum. Human embryonic stem cell line H9 (kindly provided by Professor Qinghuai Liu, Nanjing Medical University) was cultured in Essential 8 (E8) medium (Invitrogen) on Vitronectin (VTN-N)-coated plates. Mesenchymal progenitor cells (MPCs) were derived from the human embryonic stem cell line H9 (hESC H9) using the STEMdiff™ Mesenchymal Progenitor Kit (Stemcell, 05240) both of cell lines were incubated at 37 °C with 5% CO_2_.

### Three-dimensional (3D) retinal organoid differentiation from hESCs

The differentiation of human 3D retinal organoids was based on the differentiation protocol reported by the research of Sasai and David Gamm and further optimized [17, 18].

Retinal organoids were derived from the human embryonic stem cells (hESCs). Initially, hESCs were maintained on Vitronectin (VTN-N) matrix with E8 medium. When the confluence of hESCs reached 70%, Dispase (2 mg/mL) was used to dissociate to harvest embryoid bodies (EBs). And within the next four days, the culture medium was gradually transitioned from E8 medium to neural induction medium (NIM: DMEM/F12, [1:1], 1% N2 supplement, 1% MEM non-essential amino acids, and 2 mg/mL heparin sulfate). On the sixth day, 50 ng/mL BMP4 recombinant protein was introduced to the NIM medium. Subsequently, on the seventh day, NIM medium supplemented with 10% FBS was employed to facilitate the adherent growth of EBs. After 24 h, the medium was refreshed and subsequently half-replaced regularly. On the sixteenth day, EBs were lifted to obtain retinal organoids and retinal differentiation medium (RDM: DMEM/F12[3:1], 2% B27 supplement, 1% MEM non-essential amino acids, 1% penicillin-streptomycin) was employed for further culture. Starting from day 30, the RDM medium was enriched with 10% FBS, 100 μM taurine, 2 mM GlutaMAX, and 0.5 μM retinoic acid (abbreviated as RDM+3) for the long-term culture of retinal organoids.

### Lentiviral transfection

We employed two kinds of third-generation lentiviral vectors, a non-targeting human genome and another targeting hPHLDA1 knockdown, to infect developing retinal organoids. The maps of lentiviral vectors are provided in Supplementary Fig. 1. Human retinal organoids with similar sizes and morphological structures were selected and randomly divided into groups of 4–5. Each retinal organoid was transfected with 1 × 10^6^ viral transduction units in RDM + 3 medium supplemented with 5 µg/ml polybrene. After 24 h, the virus-containing medium was removed, and the organoids were maintained under normal culture conditions for 1 week before evaluation. Additionally, Y79 cells from the RB cell line were seeded into a six-well plate at a density of 2 × 10^6^ cells per well, and the lentivirus was diluted to 2 × 10^7^ viral transduction units per milliliter in RPMI 1640 medium. Following a 24-hour transfection period, the virus-containing medium was removed, and the cells were cultured under normal conditions. The cells were harvested 3 days post-infection, and the knockdown of target genes was evaluated by RT-qPCR and Western blot analysis.

### Immunofluorescence and imaging

Human retinal organoids were fixed in 4% paraformaldehyde for 30 min at 4 °C, washed with PBS, dehydrated in sucrose concentrations of gradients 6.25-12.5%-25% overnight at 4 °C, embedded in OCT, and sectioned (5 μm thickness) for storage at -80 °C. The entire immunofluorescence staining is performed at room temperature. Sections were incubated with blocking buffer(0.5% Triton X-100/PBS, 1% BSA)for 1 h, incubated with primary and secondary antibodies for 2 h for each, counterstained with DAPI(1:1000)washed, and finally mounted.

Antibody details are in Appendix Table-2. Confocal images were quantified using FIJI-ImageJ, comparing immunostaining intensity to DAPI values.

### RT-qPCR

Total RNA was extracted using TRIzol™ Reagent (Invitrogen, 15,596,026), and 1 µg was reverse-transcribed to cDNA using HiScript III RT SuperMix for qPCR (Vazyme, R323-01). Use NCBI-Primer-BLAST to design RT-qPCR primers, and use the two-step method of AceQ Universal SYBR qPCR Master Mix (Vazyme, Q511-03) for RT-qPCR detection to obtain the Ct value. Relative gene expression was calculated using 2^−△△Ct^ method, normalized against β-actin.

### Western blot

Cells were lysed using RIPA lysis buffer (50 mM Tris (pH 7.4), 0.1% SDS) and boiled for 10 min at 105 ℃. Whole cell lysates were quantified via BCA kit (Thermo Fisher Scientific, 23,252) before mixing with 5×SDS loading buffer. The prepared samples were separated by SDS-PAGE, and the electrophoretically separated bands were transferred from the gel to PVDF membrane (Millipore, IPVH00010). Membranes were then blocked with 5% skim milk (Biofroxx, 1172GR500) at room temperature for 1 h and incubated with primary antibody at 4 °C overnight. After TBST washing, the membranes were incubated with horseradish peroxidase (HRP)-labeled secondary antibody at room temperature for 1 h. ECL luminescence solution (Millipore, WBKLS0100) and Tanon5200 fully automated chemiluminescence image analysis system was used to obtain bands and ImageJ was used for quantitative analysis.

### TUNEL staining

The presence of DNA strand breaks was detected using fluorescent terminal dUTP nick end labeling (TUNEL; Serologals Corporation, Norcross, GA, USA) in the retinal organoids’ sections according to the manufacturer’s instructions. The number of apoptotic cells was counted by FIJI-ImageJ.

### CUT&Tag

The Hyperactive Universal CUT&Tag Assay Kit for Illumina (Vazyme Biotech, TD903) was used for CUT&Tag analysis. The raw reads of CUT&Tag were trimmed to 40 bp, and low-quality reads were removed using Trimmomatic v0.32. Paired reads were aligned to the human genome (version hg38) using Bowtie2 v2.3.4.2 with the parameters: “-X 2000 -no-discordant –no-contain”. Reads with a mapping quality (MAPQ) below 10 and PCR duplicated reads were filtered out using Samtools and Picard. SCEAR was used to call CUT&Tag peaks with the parameters: “--broad --broad-cutoff 0.1 -B –SPMR”. The fold enrichment for the peaks compared to a random Poisson distribution (with lambda) had to be greater than 10. The normalized signals of CUT&Tag were represented as the fold change of the treatment over the lambda control (whole genome) using macs2 bdgcmp and converted to BigWig format using bedGraphToBigWig. Peaks were annotated using chipseeker.

### RNA-seq analysis

The paired-end clean reads were aligned to the reference genome using STAR. The read counts for each gene were quantified using featureCounts. DESeq2 was employed for differential expression analysis, and the resulting p-values were adjusted using the Benjamini and Hochberg’s approach to control the false discovery rate. Genes with |log2 (Fold Change) | > 1 & adjusted P value < 0.05 were assigned as differentially expressed. GO and KEGG enrichment analysis of differentially expressed gene sets were implemented by the topG.

### Retinal organoid cell dissociation for scRNA-seq

We selected 4–5 retinal organoids with similar structural layering for experiments at the rod development time point, treated with Accutase for 30 min at 37 °C to dissociate into single-cell suspensions, and then filtered through a 40 μm cell strainer, and finally resuspend the cells in PBS containing 0.04% bovine serum albumin. ScRNA-seq libraries were prepared using the Single Cell Gene Expression 3’V2 Kit (10×Genomics, Pleasanton, CA, USA) following the manufacturer’s protocol. Briefly, single cells were distributed onto latex gel beads (GEM) within a Chromium instrument, followed by cell lysis, reverse transcription, cDNA amplification, and library construction. All libraries were sequenced on the Illumina HiSeq 2500 platform.

### ScRNA-seq analysis

#### Preprocessing of scRNA-seq data

We demultiplexed and aligned the raw scRNA-seq data to the human reference genome (GRCh38) using Cell Ranger (version 3.1) with default parameters. The expression level of each transcript was determined using UMI and transcripts were assigned to cells based on barcode. The genes filtered by the software were then used for subsequent analyses.

#### Processing and cell-type annotation of human retinal organoids scRNA-seq data

Briefly, different cell markers were used to annotate cell types. Principal component analysis (PCA) was performed on the integrated data to reduce dimensionality, and a k-nearest neighbor graph (k = 30) was constructed based on Euclidean distance in the salient PC space. Different cell clusters were identified using a Louvain-Jaccard graph-based method and labeled based on marker genes and cell type annotation from a previous study [16].

#### Identification of differentially expressed genes in scRNA-seq data sets

Model-based single-cell transcriptomics (MAST) [19]analysis was used to identify DEGs for each cell type. The identified DEGs were then tested against the asymptotic chi-square null distribution. Genes with FDR correct *P* < 0.05 were considered to be differentially expressed.

#### Gene ontology analysis of DEGs

Gene ontology (GO) analysis was performed using clusterProfiler (version 4.0) [20]and visualized using the ggplot2 R package (https://github.com/tidyverse/ggplot2). GO terms or pathways were considered enriched at adjusted *P* < 0.05.

### Statistical analysis

All experiments were performed at least three times. Statistical analysis of data between different groups used GraphPad Prism 8 software for two-tailed unpaired t-test. Data were presented as mean ± SEM. An unpaired two-tailed Student’s t-test was used to determine significance, denoted by ns, not significant; *p < 0.05; ***p < 0.001 and ****p < 0.0001.

## Results

### Lentiviral vectors disrupt the normal differentiation of retinal progenitor cells into photoreceptor cells

**Fig. 1:**
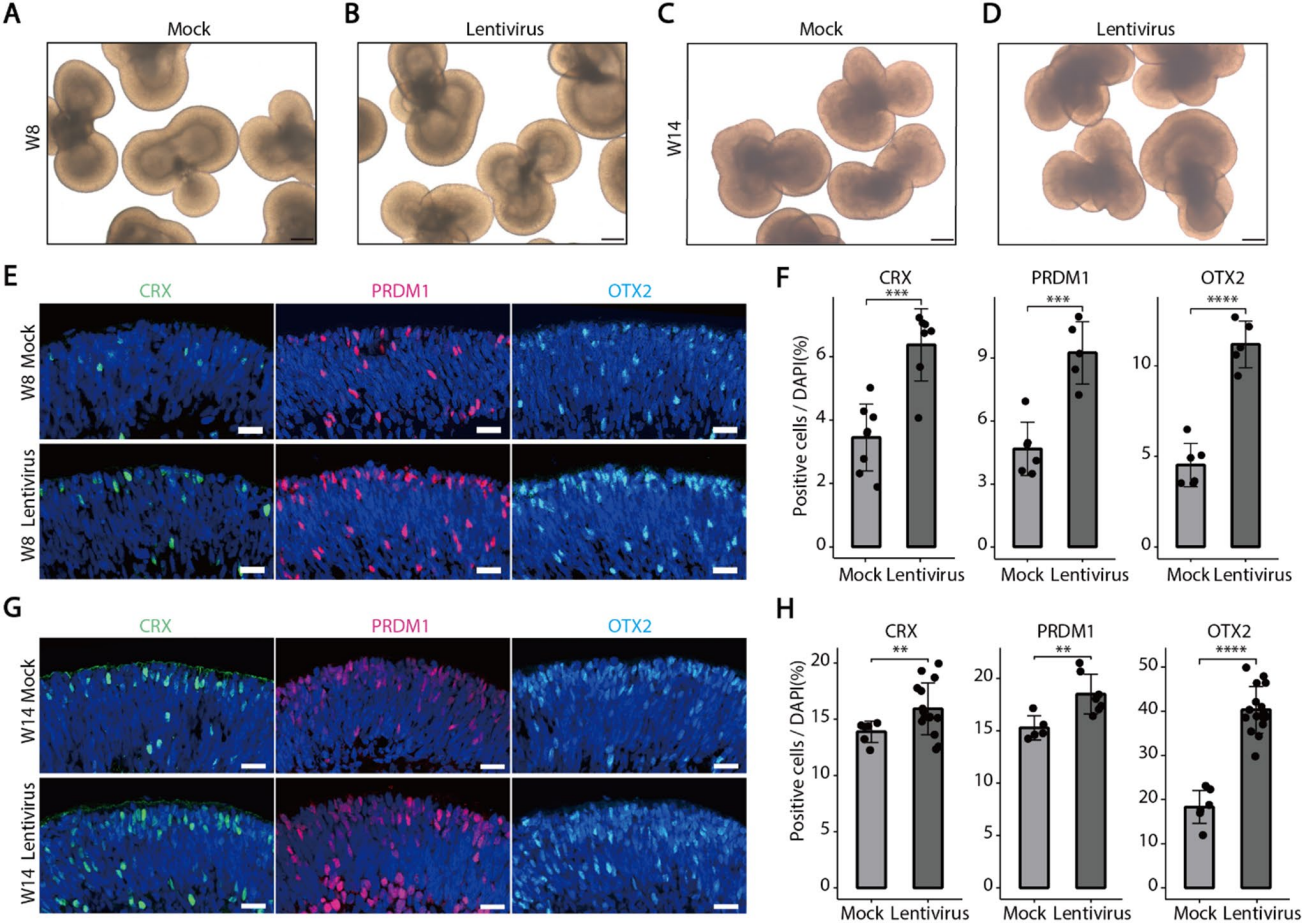
Lentiviral vectors disrupt the normal differentiation of retinal progenitor cells into photoreceptor cells. **A**-**D** Bright-field images of retinal organoids at both early and late stages in mock and lentivirus-infected groups. Scale bars, 500 μm. **E**-**H** In immunofluorescence images, the early and late stages of retinal organoids exhibited a significant increase in the numbers of CRX^+^, PRDM1^+^, and OTX2^+^ photoreceptor cells within the lentivirus-infected group compared to the mock group. Scale bars, 20 μm

To further assess the impact of viral infection on retinal development, we infected retinal organoids with an empty lentiviral vector (Supplementary Fig. 1A). Given that different retinal cell types have distinct developmental windows, we performed lentiviral infections (MOI = 10) at both early (week 7) and late (week 13) developmental stages. One week post-infection, we observed that lentiviral infection did not cause any significant morphological or structural changes in the retinal organoids (Fig. 1A-D). Further analysis using immunofluorescence staining revealed that lentiviral infection led to an increase in neurogenic RPCs (ASCL1^+^ cells) (Supplementary Fig. 2G-H), cone photoreceptors (OTX2^+^, CRX^+^, PRDM1^+^ cells at week 8), and rod photoreceptors (OTX2^+^, CRX^+^, PRDM1^+^ cells at week 14) (Fig. 1E-H). However, other cell types remained unaffected, including retinal ganglion cells (RGCs; ISLET1^+^ cells), horizontal cells (HCs; ONECUT2^+^ cells), and amacrine cells (ACs; TFAP2A^+^ cells) (Supplementary Fig. 2A-F). These results suggest that lentiviral vectors can cause to aberrant differentiation of photoreceptor cells in retinal organoids.

### PHLDA1 mediates the effects of lentiviral infection on the specification of retinal progenitor cells into photoreceptor cells

**Fig. 2:**
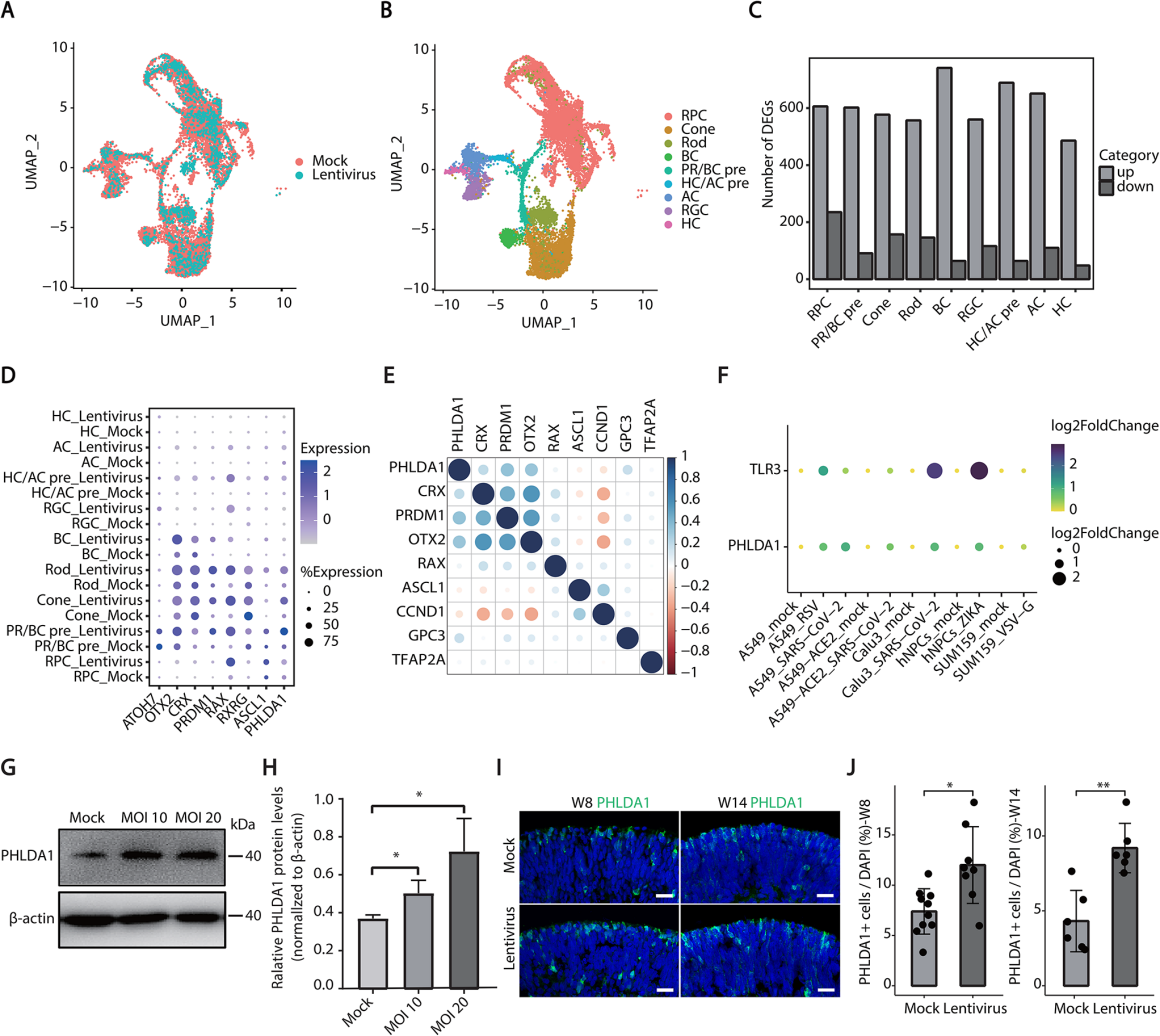
Lentiviral infection induces significant up-regulation of *PHLDA1* expression. **A**-**B** UMAP analysis of scRNA-seq comparing the lentivirus-infected group to the mock group in retinal organoids (W13), including 9 distinct cell types: RPC, (retinal progenitor cell); Cone/Rod, (photoreceptor cell); BC, (bipolar cell); PR/BC pre, (photoreceptor cell/bipolar cell precursor); HC/AC pre, (horizontal cell/amacrine cell precursor); AC, (amacrine cell); RGC, (retinal ganglion cell); HC, (horizontal cell). **C** Differential gene expression patterns were observed in the lentivirus-infected cells of human retinal organoid. **D** Dot plot showing the up-regulation of photoreceptor fate-determining genes in retinal organoids caused by lentiviral infection. **E** Gene correlation analysis shows the key photoreceptor fate-determining genes such as *CRX*, *PRDM1*, and *OTX2* exhibited a high positive correlation with PHLDA1 **F** Transcriptome analysis showing significantly up-regulation of PHLDA1 caused by the transfections of SARS-CoV-2, RSV, ZIKA, and VSV-G viruses compared with the corresponding mock group. **G-H** Western blot analysis of PHLDA1 in Y79 cells at varying multiplicity of infection (MOI = 10, MOI = 20). **I-J** The number of PHLDA1+ cells was significantly up-regulated in the lentivirus group compared to the mock group in both early (week 8) and late (week 14) developmental stages? human retinal organoids. Scale bars, 20 μm

To unravel the molecular mechanisms responsible for the abnormal development of photoreceptor cells induced by lentiviral vectors, we performed single-cell RNA sequencing on both unaffected (Mock) and lentiviral vectors infected (Lentivirus) retinal organoids, identifying transcriptional profiles of 14,086 cells totally. Through cell clustering and cell type annotation, we identified 9 retinal cell types, including proliferative RPCs, cones, rods, bipolar cells, photoreceptor /bipolar precursors, horizontal /amacrine precursors, amacrine cells, retinal ganglion cells, horizontal cells, etc. (Fig. 2A-B). Differential gene expression (DGE) analysis further revealed significant changes in the gene expression patterns of all retinal cell types following lentiviral infection of retinal organoids (Fig. 2C, Supplementary Fig. 3). To explore mechanisms underlying the abnormal differentiation of photoreceptor cells induced by lentiviral vectors, we first analyzed the expression patterns of photoreceptor fate-determining genes following lentiviral infection, and found an up-regulation of photoreceptor fate-determining genes (*OTX2*, *CRX*, *PRDM1*, *RAX*, *RXRG* and *PHLDA1*, etc.) [21–23] (Fig. 2D, Supplementary Fig. 4). We further observed a correlation between *PHLDA1* and other critical genes such as *CRX, OTX2, and PRDM1* via gene correlation analysis in all cells (Fig. 2E). By performing lentiviral infection on retinoblastoma cell line Y79, we found that the MOI of lentiviral vectors was positively correlated with the protein expression level of PHLDA1 (Fig. 2G-H). Subsequently, we confirmed that lentiviral infection significantly increased the number of PHLDA1^+^ cells in retinal organoids through immunofluorescence staining (Fig. 2I-J). We next collected transcriptome data from cells infected with various viruses including SARS-CoV-2, RSV, ZIKA, and VSV-G. The results showed consistent up-regulation of PHLDA1 across different cell types upon different viral infections, suggesting *PHLDA1* is a key intracellular regulator upon viral infections (Fig. 2F**)**. In summary, our results indicate that PHLDA1 may play an important role in driving abnormal differentiation of photoreceptor cells during lentiviral infection.

**Fig. 3:**
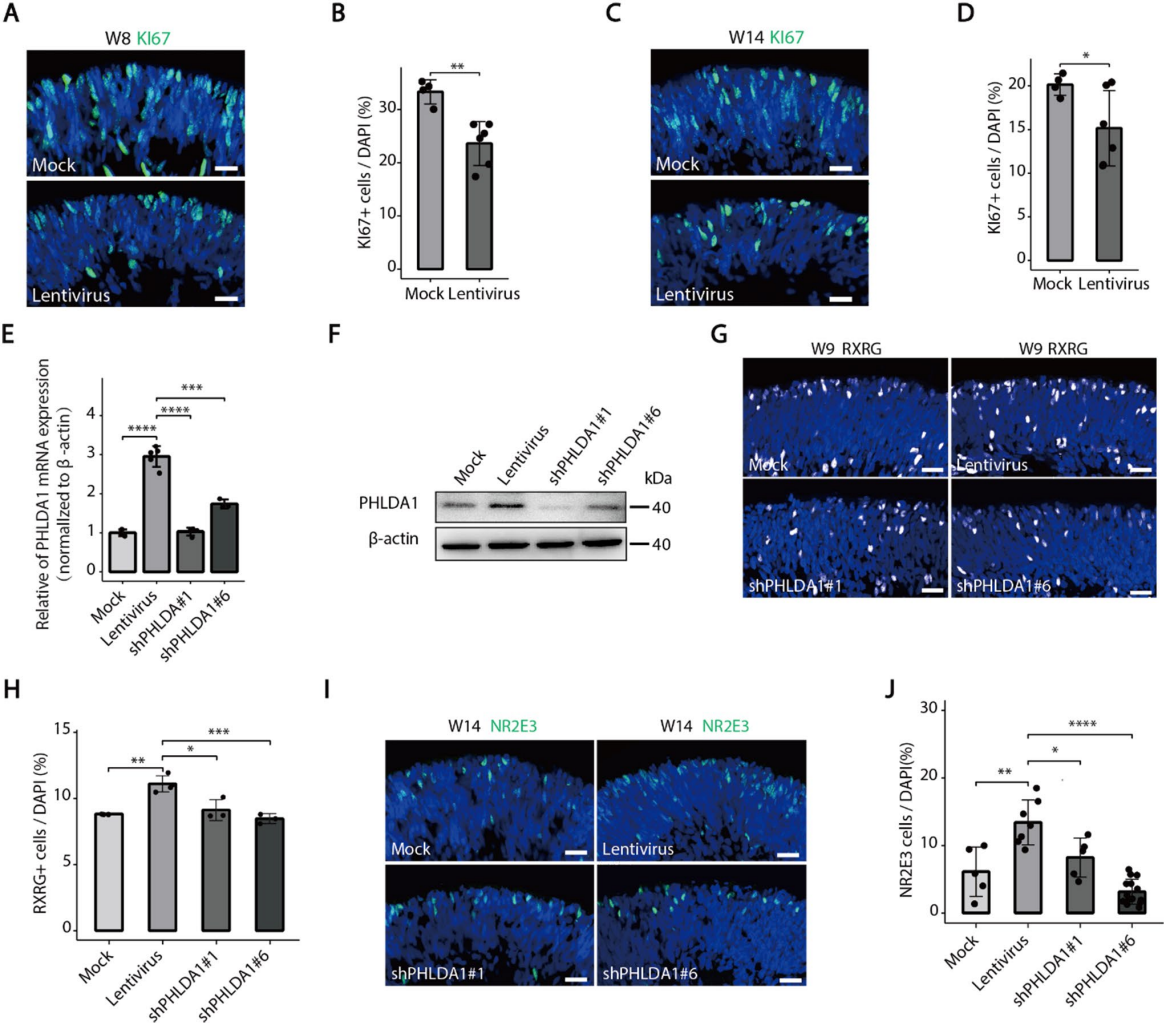
*PHLDA1*-mediated the effects of lentiviral infection on the specification of retinal progenitor cells into photoreceptor cells. **A-D** Immunofluorescence revealed that in both early (week 8) and late (week 14) developmental human retinal organoids, lentiviral infection resulted in a reduction of KI67+ retinal progenitor cells compared with the mock control group. Scale bars, 20 μm. **E-F** RT-qPCR and Western blot analysis of PHLDA1 in Y79 cells in the lentivirus group and shPHLDA1(KD) group. **G-H** Immunofluorescence showed that the knockdown of PHLDA1 markedly suppressed the lentivirus-induced increase in RXRG^+^ cells numbers in early-stage retinal organoids. Scale bars, 20 μm. **I-J** Immunofluorescence showed that knockdown of *PHLDA1* significantly obstructed the rise in NR2E3^+^ cells counts caused by lentiviral infection in the late stages of retinal organoids. Scale bars, 20 μm

Our previous research found that knockdown of *PHLDA1* led to an increase in proliferative RPCs numbers [21], suggesting that PHLDA1 may promote RPC differentiation into photoreceptor cells. In this study, we conducted cell proliferation and apoptosis assays in retinal organoids infected with lentiviral vectors and found that lentiviral infection led to a decrease in RPCs (MKI67^+^) and an increase apoptotic cells (TUNEL+, from 0.45% to 1.07% for W8, and from 0.72% to 1.17% for W14, respectively) (Fig. 3A-D, Supplementary Fig. 5A-D), but the number of apoptotic cells is significantly lower than the decreased proliferating cells. Therefore, these results indicate that the increase in the number of photoreceptor cells may be due to abnormal RPC differentiation caused by lentiviral infection. Lentiviral vectors were employed to mediate PHLDA1 knockdown in Y79 cells. Further, RT-qPCR and Western blot analyses revealed that this approach significantly decreased PHLDA1 expression levels initially upregulated by the lentiviral vectors (Fig. 3E-F). Additionally, the lentiviral vector-mediated knockdown of PHLDA1 markedly inhibited the rise in photoreceptor cell numbers (RXRG+, NR2E3+, CRX+, OTX2+) typically triggered by lentiviral infection in retinal organoids (Fig. 3G-J, Supplementary Fig. 6–7). These results indicate that the interference with the fate of photoreceptor cells affected by lentiviral vectors is mainly through the up-regulation of PHLDA1, thereby triggering the fate deviation of RPCs towards photoreceptor cells.

### PHLDA1 regulates the expression of key transcription factors that determine photoreceptor cell fate

Structural analysis of PHLDA1 revealed the presence of QQ and PQ domains, which are commonly found in transcription factors, implicating a potential involvement of *PHLDA1* in regulating gene expression [24, 25]. Based on these findings, we hypothesized that *PHLDA1* may be involved in regulating the expression of genes crucial for determining the fate of photoreceptor cells during their differentiation process. To examine this hypothesis, we first performed immunofluorescence to determine the subcellular localization of PHLDA1. Our results indicated that PHLDA1 was present in both the cytoplasm and the nucleus (Supplementary Fig. 8A–C) [26], suggesting a possible role in gene regulation.

**Fig. 4:**
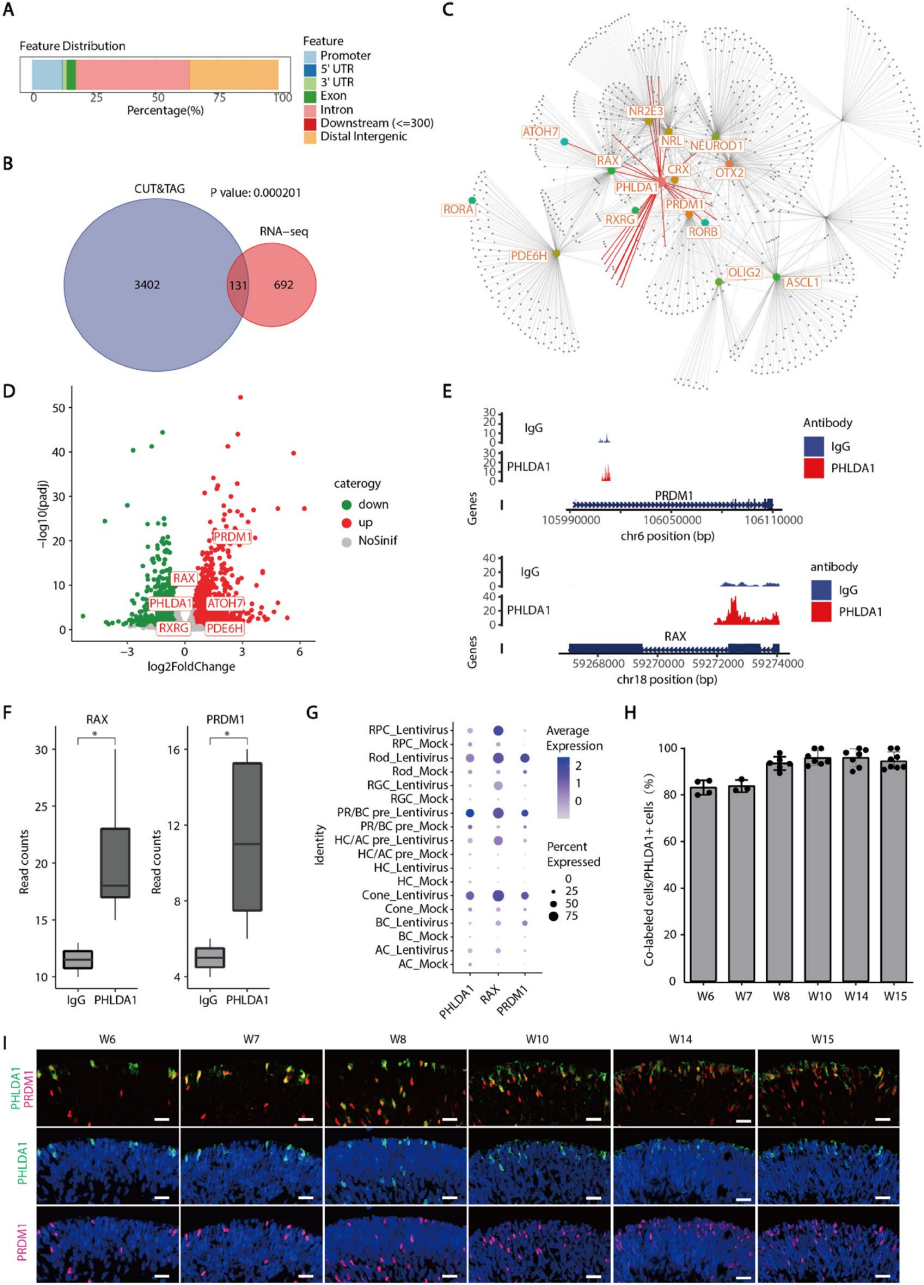
*PHLDA1* possesses transcription factor activity. **A** Bar graph depicted the genomic distribution of *PHLDA1* binding sites. **B** Venn diagram compared the genes identified by CUT&Tag and RNA sequencing as differentially expressed between the lentivirus-infected and control (mock) groups. **C** Gene network diagram showed key genes for photoreceptor cell fate determination with *PHLDA1* binding sites. **D** The volcano plot showed that compared with the mock group, several crucial genes associated with photoreceptor cell fate determination were notably up-regulated following the lentiviral infection of Y79 cells. **E** Trackplot showed that *PHLDA1* could bind to the *PRDM1* intergenic region, as well as to the *RAX* promoter region. **F** The bar-plot showed the difference in read counts between IgG and PHLDA1. **G** Single-cell transcriptome analysis revealed that the expression of *PRDM1* and *RAX* in photoreceptor cells was significantly up-regulated in the lentivirus-infected group compared to the mock group. **H-I** Immunofluorescence staining images displayed the co-expression of PHLDA1 with PRDM1 in human retinal organoids at different development stages. Scale bars, 20 μm

To further elucidate the role of *PHLDA1* in gene expression, we established three stable Y79 cell lines following lentiviral infection and performed CUT&Tag experiments coupled with RNA-seq. Our analyses revealed that *PHLDA1* was bound to DNA elements and influenced the expression of target genes. By integrating CUT&Tag with gene regulatory network analysis, we identified the potential role of *PHLDA1* in mediating photoreceptor differentiation. This analysis showed that *PHLDA1* was bound to DNA regulatory elements of key genes involved in determining the fate of photoreceptor cells, including *RAX, PRDM1, NR2E3*, and *NRL* (Fig. 4A-C). Correspondingly, RNA-seq data showed the significant up-regulation of genes crucial for determining the fate of photoreceptor cells, such as *RAX, PRDM1, ATOH7, RXRG*, and *PDE6H* following lentiviral infection. (Fig. 4D). We further investigated the binding sites of *PHLDA1* in these genes, discovering they bind to the intronic and intergenic regions of *PRDM1*, as well as the promoter region of *RAX* (Fig. 4E-F**)**.

Finally, we validated these findings by comparing single-cell RNA-seq data from lentivirus-infected retinal organoids with those from mock control. Our results indicated that lentiviral infection induced elevated expression of *PHLDA1* in photoreceptor cells, accompanied by a significant up-regulation of *PRDM1* and *RAX* expression. Moreover, PRDM1 showed up-regulation in different cells transfected with different viruses, consistent with PHLDA1 (Fig. 4G, Supplementary Fig. 9). Subsequently, through immunofluorescence staining, we found that PHLDA1 is predominantly co-expressed with PRDM1 and RAX at different developmental stages in retinal organoids (Fig. 4H-I, Supplementary Fig. 10A-B). And we also found that lentivirus vector infection significantly increased the co-location of PHLDA1 and RAX, and knockdown PHLDA1 reduced that, suggesting PHLDA1 can regulate the expression of RAX in retinal organoids (Supplementary Fig. 10C-D). Based on these findings, we hypothesize that PHLDA1 may possess transcriptional activity and disrupt the specialization of photoreceptor cells during lentiviral infection by activating the expression of RAX and PRDM1.

### Reducing PHLDA1 expression neutralized the lentiviral vector-induced disturbance in the fate specification of retinal progenitor cells

**Fig. 5:**
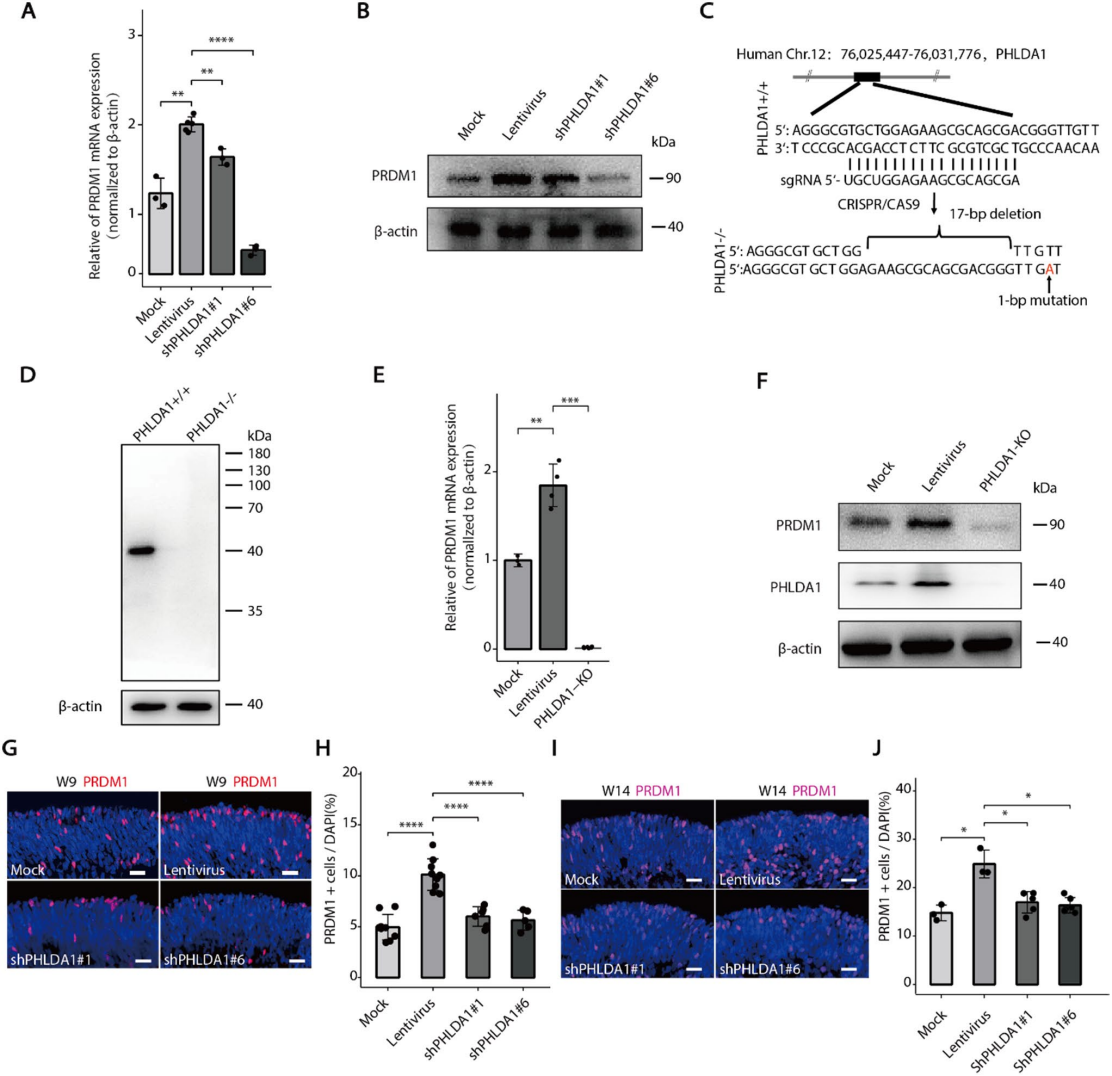
Lentiviral infection activates PHLDA1-PRDM1 and promotes photoreceptor cell specialization. **A-B** RT-qPCR and Western blot analyses demonstrated that silencing PHLDA1 in Y79 cells markedly suppressed the lentivirus-induced up-regulation of PRDM1 expression. **C-D** Details on the specific regions knocked out and the mutation sites of PHLDA1 in this study. **E-F** RT-qPCR and Western blot demonstrated that knockout of PHLDA1 significantly reduced the lentivirus-mediated up-regulation of PRDM1 expression in Y79 cells. **G-J** Immunofluorescence imaging showing the PRDM1^+^ photoreceptor cells in W9 and W14 retinal organoids across different groups: control (Mock), lentivirus-infected(Lentivirus), and PHLDA1 knockdown (shPHLDA1#1, shPHLDA1#6). Scale bars, 20 μm

Previous studies have demonstrated that *PRDM1* (encoding for BLIMP1 transcription factor) plays a pivotal role in inhibiting the differentiation of photoreceptor/bipolar precursors into bipolar cells while promoting their differentiation into photoreceptors [27]. Building upon this foundation, we hypothesized that the PHLDA1-PRDM1 axis was critically involved in regulating the aberrant differentiation of photoreceptors. To validate this hypothesis, we initially investigated the influence of *PHLDA1* on *PRDM1* expression in the Y79 cell line. Through lentiviral vector-mediated shRNA, we successfully knocked down *PHLDA1*, which subsequently led to a significant reduction in PRDM1 expression (Fig. 5A-B). Subsequently, we further knocked out *PHLDA1* in Y79 cells, with the results demonstrating that *PHLDA1* knockout significantly inhibited the up-regulated expression of PRDM1 induced by lentiviral infection (Fig. 5C-F, Supplementary Fig. 11). These findings suggest that PHLDA1 is likely to modulate the expression of PRDM1 in photoreceptor precursors. Additionally, we performed lentivirus-mediated knockdown of *PHLDA1* in retinal organoids during the developmental stages of cones and rods, respectively, and found a significant reduction in the number of PRDM1^+^ cells (Fig. 5G-J). Notably, we also observed an overproduction of photoreceptor cells, as evidenced by an increased count of PRDM1^+^ cells at both developmental stages, following lentivirus infection. Interestingly, the number of PRDM1^+^ cells post-PHLDA1 knockdown was comparable to that in the mock group (Fig. 5G-J), implying that PHLDA1 may play a dominant role in modulating photoreceptor production during lentiviral infection. In summary, our data indicate that PHLDA1 is a key regulator in the specification into photoreceptors within RPCs, and its knockdown seems to mitigate the effects of lentiviral vectors on RPCs fate determination.

## Discussion

In this study, we found that lentiviral vectors’ stimulation led to abnormal development of photoreceptor cells in human retinal organoids. Further analysis of single-cell transcriptome data revealed that the expression of the photoreceptor cell differentiation regulatory gene *PHLDA1* was up-regulated after virus infection. Furthermore, through CUT&Tag experiments, we validated the transcription activation properties of *PHLDA1* and discovered its ability to regulate the expression of key transcription factors involved in photoreceptor cell fate determination, such as *RAX* and *PRDM1*. This regulatory function of PHLDA1 promotes the differentiation of RPCs into photoreceptor cells. We identified the phenotypic and molecular mechanisms of abnormal development of photoreceptor cells in the human retina caused by lentiviral vectors.

By analyzing transcriptome data from various cell types infected with different viruses, we found that PHLDA1 was commonly upregulated (Fig. 2F), suggesting that PHLDA1 up-regulation may be a common response of various cell types to viral infection. Several potential molecular mechanisms may contribute to virus-induced PHLDA1 up-regulation, such as ER stress, autophagy and inflammation. Firstly, studies have shown that PHLDA1 expression is significantly upregulated following treatment with various ER-stress-inducing agents, including dithiothreitol (DTT), thapsigargin, tunicamycin, and farnesol, whereas agents that attenuate ER stress, such as salubrinal (a selective eIF2⍺ inhibitor) and intracellular calcium chelator BAPTA, mitigate the up-regulation of PHLDA1 [28–30]. Secondly, autophagy is considered as a defense strategy in organisms and plays an antiviral function. Upon viral infection, host cells rapidly initiate autophagy to degrade viral particles or virus components. Studies have shown that Rapamycin, a well-known autophagy activator, upregulates PHLDA1 expression in T-47 mammary cells [31]. Thirdly, in immune cells, such as microglia [32] and bone marrow-derived macrophages [33], lipopolysaccharide (LPS) treatment can increase the expression level of PHLDA1, which was mediated by the TLR2/4 signaling pathway. PHLDA1 could activate NF-κB signaling to promote the expression of pro-inflammatory cytokines, including TNF-α, IL-1β, iNOS, and COX-2. Some studies have revealed how lentiviral infection causes the above pathways. For example, the envelope protein VSV-G is used to package viruses, but research has found that it can activate signaling pathways through the TLR4 and CD14 receptors, triggering the production of type I interferons [34]. Recent studies have identified that P53 is the key regulatory factor that regulates the PHLDA1 expression by recruiting CBP and P300 to its promoter region [35], which may potentially serve as a hub of virus-induced PHLDA1 up-regulation. P53 can be activated by viral infection through various pathways. For example, cellular stress (such as oxidative stress and endoplasmic reticulum stress) can also promote P53 phosphorylation [36]. Inflammatory factors induced by viral infection (such as interferons and TNF-α) can further promote P53 activation and expression [37, 38]. Moreover, viral DNA or RNA can also induce P53 activation. For instance, HIV RNA can be recognized by the intracellular RIG-I and MDA5 receptors to promote type I interferon production, which promotes P53 activation [39, 40]. In conclusion, viral infection can induce PHLDA1 up-regulation through multiple pathways, including ER stress, interferons and pro-inflammatory, and these pathways finally activate P53, which leads to increased PHLDA1 expression.

In this study, we found that PHLDA1 possessed potential transcriptional regulatory activity as demonstrated by CUT&Tag experiments revealing its ability to bind to the RAX promoter region and the PRDM1 intronic region. Knockdown or knockout of PHLDA1 significantly reduced the expression levels of both RAX and PRDM1. Previous research has established the crucial role of RAX in photoreceptor cell development. RAX directly binds and activates the EELPOT enhancer on the OTX2 gene, thereby activating OTX2 transcription. Conditional knockout of RAX in photoreceptor precursors results in decreased OTX2 expression [41]. Furthermore, the absence of RAX activity in the early retinal progenitors, due to deletion by the Pax6α-Cre driver, leads to the loss of cone and late-born retinal neurons, such as rods and bipolar cells. Therefore, PHLDA1 may promote the differentiation of RPCs into photoreceptor/bipolar precursors by upregulated RAX expression. Our results also show that PHLDA1 can bind to and regulate the expression level of PRDM1, a gene known to play an important role in regulating the differentiation of OTX2^+^ cells into photoreceptor cells while inhibiting their differentiation into bipolar cells through the suppression of VSX2 expression [27]. Moreover, ectopic expression of PRDM1 in immature bipolar cells leads to their differentiation into photoreceptor cells [42]. Therefore, these findings suggest that PHLDA1 may maintain the specific differentiation of nascent OTX2^+^ cells into photoreceptor cells rather than bipolar cells by promoting PRDM1 expression. In summary, these pieces of evidence suggest that PHLDA1 potentially facilitates the differentiation of photoreceptor cells via a biphasic mechanism. Initially, it upregulates RAX in RPCs, promoting their differentiation into OTX2+ photoreceptor/bipolar precursors; subsequently, PHLDA1 enables the differentiation of OTX2+ cells into mature photoreceptors by upregulating PRDM1. Further research is required to elucidate the molecular mechanisms through which PHLDA1 mediates the photoreceptor/bipolar fate bifurcation.

As a downstream target gene of PHLDA1, it has been that PRDM1 is an important transcriptional repressor that plays multiple roles in viral infection. Additionally, viral infection can induce PRDM1 expression [43]. By binding to the IFN-β promoter, it inhibits its transcription, thereby exerting a negative feedback regulation after IFN-I induction [44, 45] and promoting viral replication. In plasmacytoid dendritic cells (pDCs), PRDM1 promotes type I interferon production and enhances the antiviral response [46]. Our findings suggest that PHLDA1 may have a role in promoting the interferon pathway in other immune systems, however, the specific functions of this pathway in other viral infection systems still require further research.

The use of lentiviral vectors in gene therapy has always been controversial [47, 48]. Our findings show that lentiviral vectors, as a tool for gene therapy, can affect the fate determination of RPCs and increase the number of photoreceptor cells, even though they do not cause significant pathological changes under traditional infections (MOI = 10). This early abnormal development of the retina resulting in changes in cell numbers may not necessarily lead to significant visual impairment in the early stages but could potentially have a delayed and cumulative effect, ultimately influencing retinal function in a mature retina. Our study has found that lentiviral infection can cause cell fate bias which warrants caution for the clinical use of lentiviral gene intervention. Clinicians need to carefully select delivery vectors and optimize clinical protocols to avoid risks and enhance social responsibility. In addition, we found that the use of lentiviral vectors can activate the PHLDA1-PRDM1 axis and affect retinal development, which provides a new reference indicator for evaluating viral vector risks or cell fate changes in other retinal development models. In our study, it was found that the use of lentiviral vectors induced up-regulation of PHLDA1 during the differentiation of photoreceptor cells. *PHLDA1* has been identified as a gene that is associated with a variety of tumors [49, 50] and as one that promotes tumor growth [51, 52]. Through the process of gene therapy for disease rescue, lentiviral vectors infection induces the up-regulation of PHLDA1 expression, which may cause some potential safety risks, including the possibility of promoting tumorigenesis and leading to adverse prognosis. These issues need to be paid attention to and solved in the application of gene therapy. Thus, long-term patient follow-up is required after gene therapy to obtain accurate information and evaluate whether there is an association between lentiviral vectors and tumor development, recurrence, and prognosis.

Clearly, our study provides evidence that lentivirus affects the specification of RPCs into photoreceptor cells by activating the PHLDA1-PRDM1 axis. This suggests that lentiviral vectors might have adverse effects in clinical applications. As of 2023, the number of HIV-infected individuals has reached as high as 39 million, with an annual infection rate of 1.5 million [53]. Currently, there is insufficient attention given to the impact of HIV infection on the retina, despite the fact that HIV can be detected in the nervous system [54, 55]. However, we observed the impact of lentiviral vectors on retinal development in a model that was highly relevant to the retina. As the number of HIV infections rises, comprehending the effects of HIV infection on fetal neural system development becomes particularly important. Mother-to-child transmission is one of the major pathways of HIV transmission, and the virus may be transmitted to the fetus through vertical transmission when an infected mother gives birth. Therefore, it is crucial to pay attention to the impact of mother-to-child transmission of HIV infection on fetal neurodevelopment. This will help to formulate corresponding prevention and intervention measures to minimize potential adverse effects.

One limitation of this study is that, although we discovered that PHLDA1 possessed potential transcriptional activation functions and could bind to the promoter region of RAX and the intronic region of PRDM1, and knockdown/knockout of PHLDA1 significantly reducing the expression levels of these genes, additional research is required to elucidate whether PHLDA1 directly binds to DNA or regulates the transcription of downstream genes through interactions with other proteins. Furthermore, while our findings indicate that viral infection can induce aberrant photoreceptor development within a short time frame (1 week), the long-term consequences on retinal development necessitate further investigation.

## Electronic supplementary material

Below is the link to the electronic supplementary material.

Supplementary Material 1

## Data Availability

We obtained the raw sequencing data of different virus infector from the NCBI Sequence Read Archive under accession number GSE147507、GSE78711 and GSE168125. ScRNA-seq data from retinal organoids have been deposited in the Gene Expression Omnibus (GEO) under accession number GSE136929. CUT&Tag and RNA sequence data from Y79 have been deposited in the Gene Expression Omnibus (GEO) under accession number GSE251752.The data and material that support the findings of this study are available from the corresponding author on reasonable request.

## Abbreviations

PHLDA1: Pleckstrin homology-like domain, family A, member 1
PRDM1: PR domain zinc finger protein 1
LCA: Leber’s congenital amaurosis
NRL: Neural retina leucine zipper
CEP290: Centrosomal protein 290
NR2E3: Nuclear receptor subfamily 2 group E member 3
OTX2: Orthodenticle homeobox 2
CRX: Cone–rod homeobox protein
RAX: Retina and anterior neural fold homeobox gene
RXRG: Retinoid X receptor gamma
SARS-CoV-2: Severe acute respiratory syndrome coronavirus 2
RSV: Respiratory syncytial virus
VSV-G: Vesicular stomatitis virus G
hESC: Human embryonic stem cell
RPCs: Retinal progenitor cells
ER: Endoplasmic reticulum
UPR: Unfolded protein response
PQ: Proline-glutamine
QQ: Polyglutamine
HIV: Human immunodeficiency virus
