# Supplementary material for: PHLDA1-PRDM1 mediates the effect of lentiviral vectors on fate-determination of human retinal progenitor cells
Source: Cell Mol Life Sci. 2024 Jul 16;81(1):305. doi: 10.1007/s00018-024-05279-z (PMC11335229; doi:10.1007/s00018-024-05279-z)
Supplement: Supplementary file 1 — Supplementary Material 1 [file 18_2024_5279_MOESM1_ESM.docx]

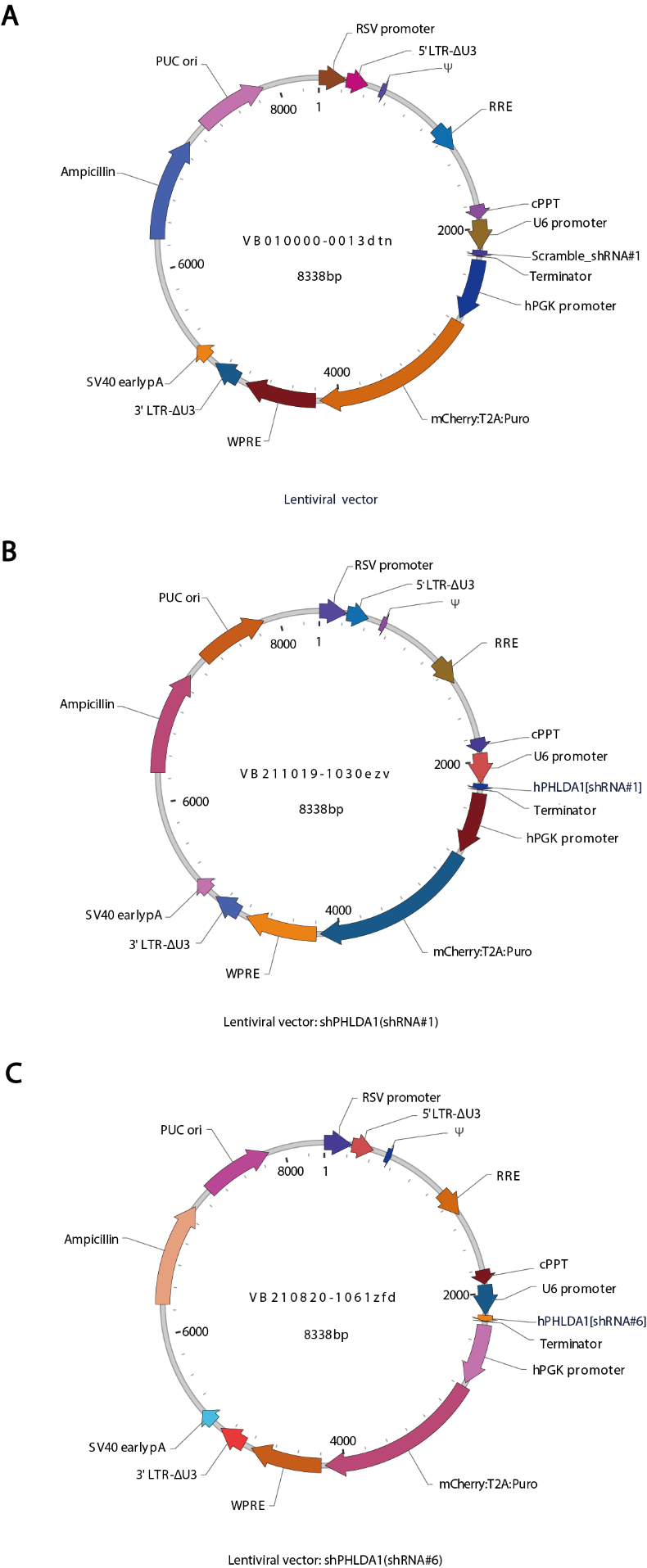


**Supplementary Fig. 1 Plasmid map of lentiviral vector-transduced retinal organoids. A** An empty lentiviral vector packed with non-targeting human shRNA. **B-C** Lentiviral vectors for targeted knockdown of PHLDA1.

**
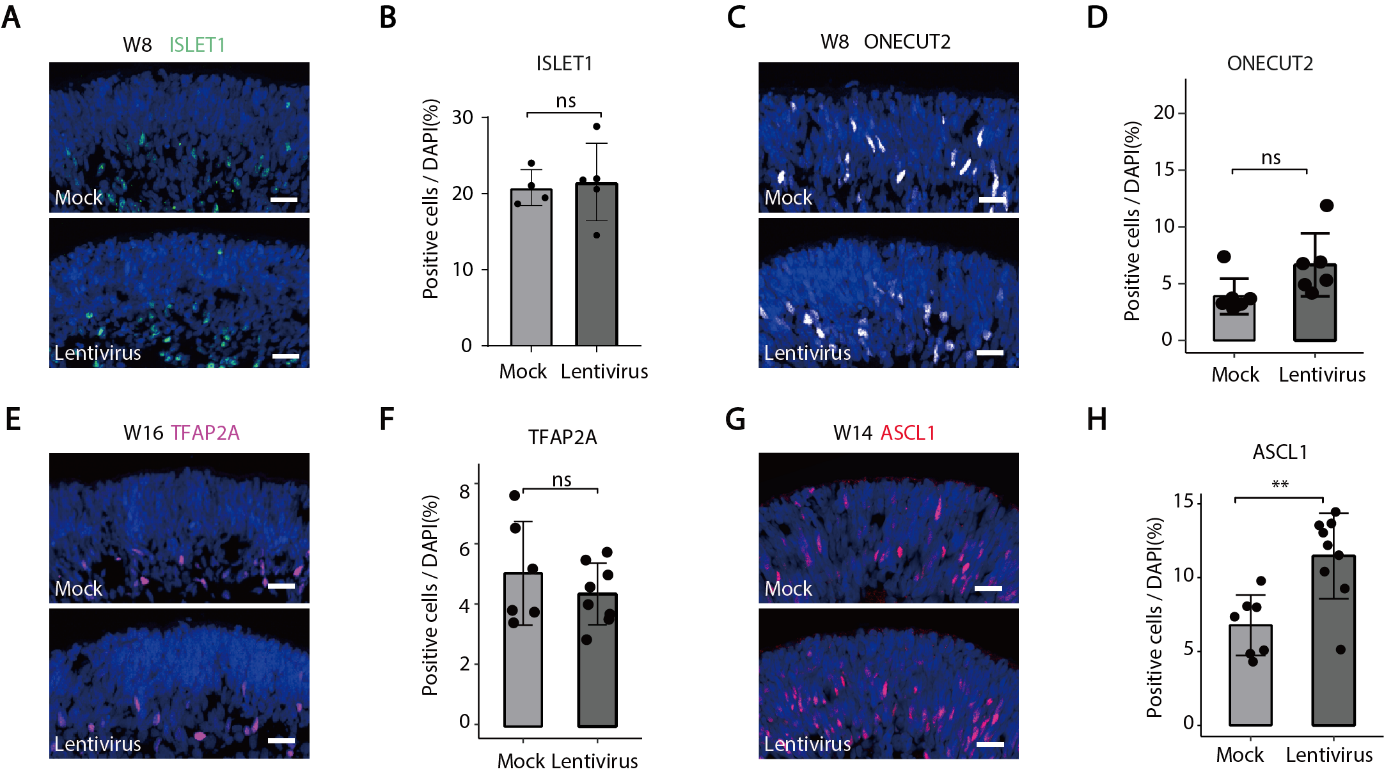
**

**Supplementary Fig. 2 The effect of lentiviral vectors infection on other retinal cell types. A-B** Lentivirus infection did not cause significant changes in the number of (ISLET1^+^) retinal ganglion cells in retinal organoids. Scale bars, 20 μm. **C-D** Lentivirus infection did not cause significant changes in (ONECUT2^+^) horizontal cells. Scale bars, 20 μm. **E-F** Lentivirus infection did not cause significant changes in (TFAP2A^+^) amacrine cells. Scale bars, 20 μm. **G-H** The numbers of (ASCL1^+^**)** neurogenic retinal progenitor cells were significantly increased after infection of lentiviral vectors. Scale bars, 20 μm.


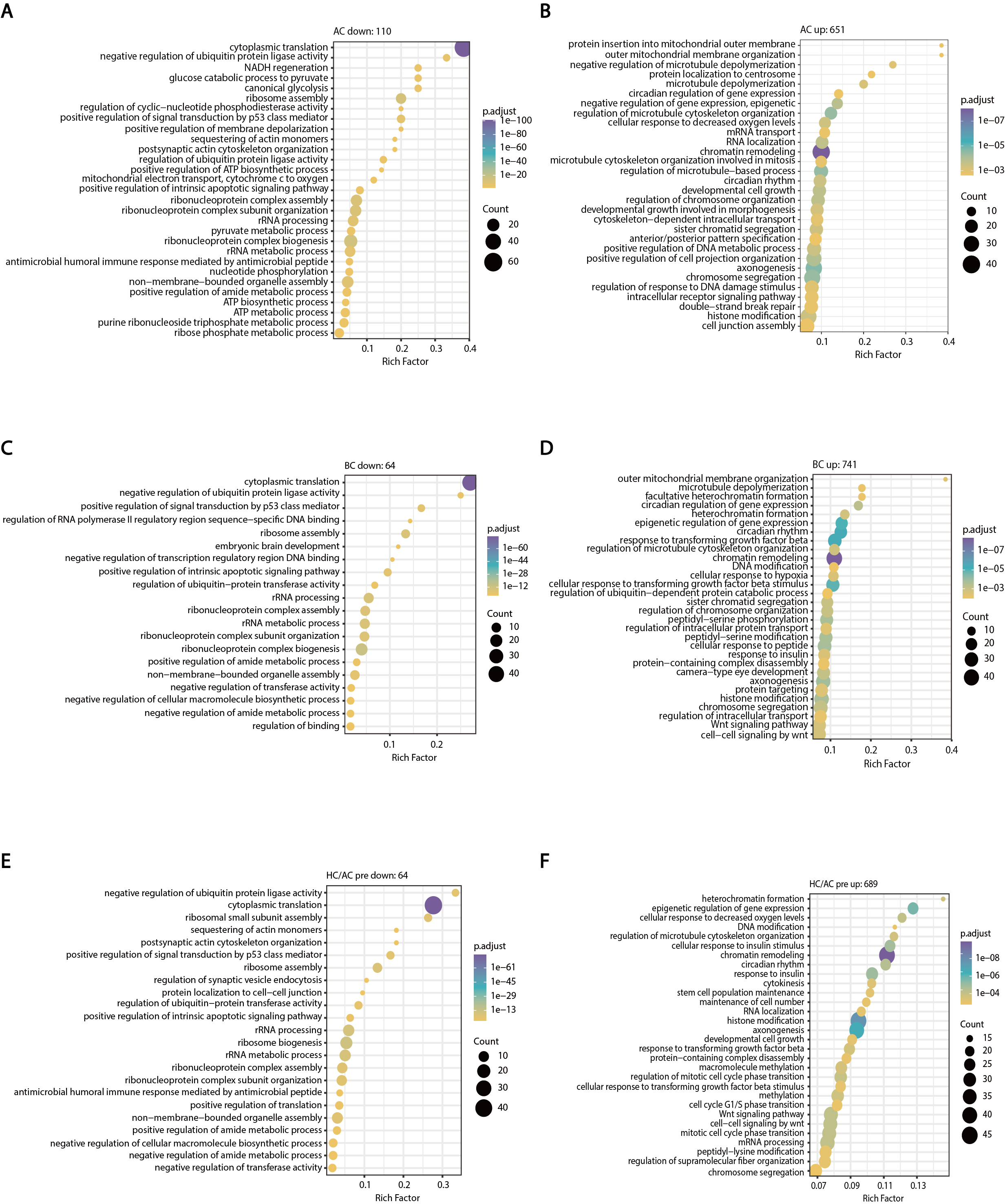


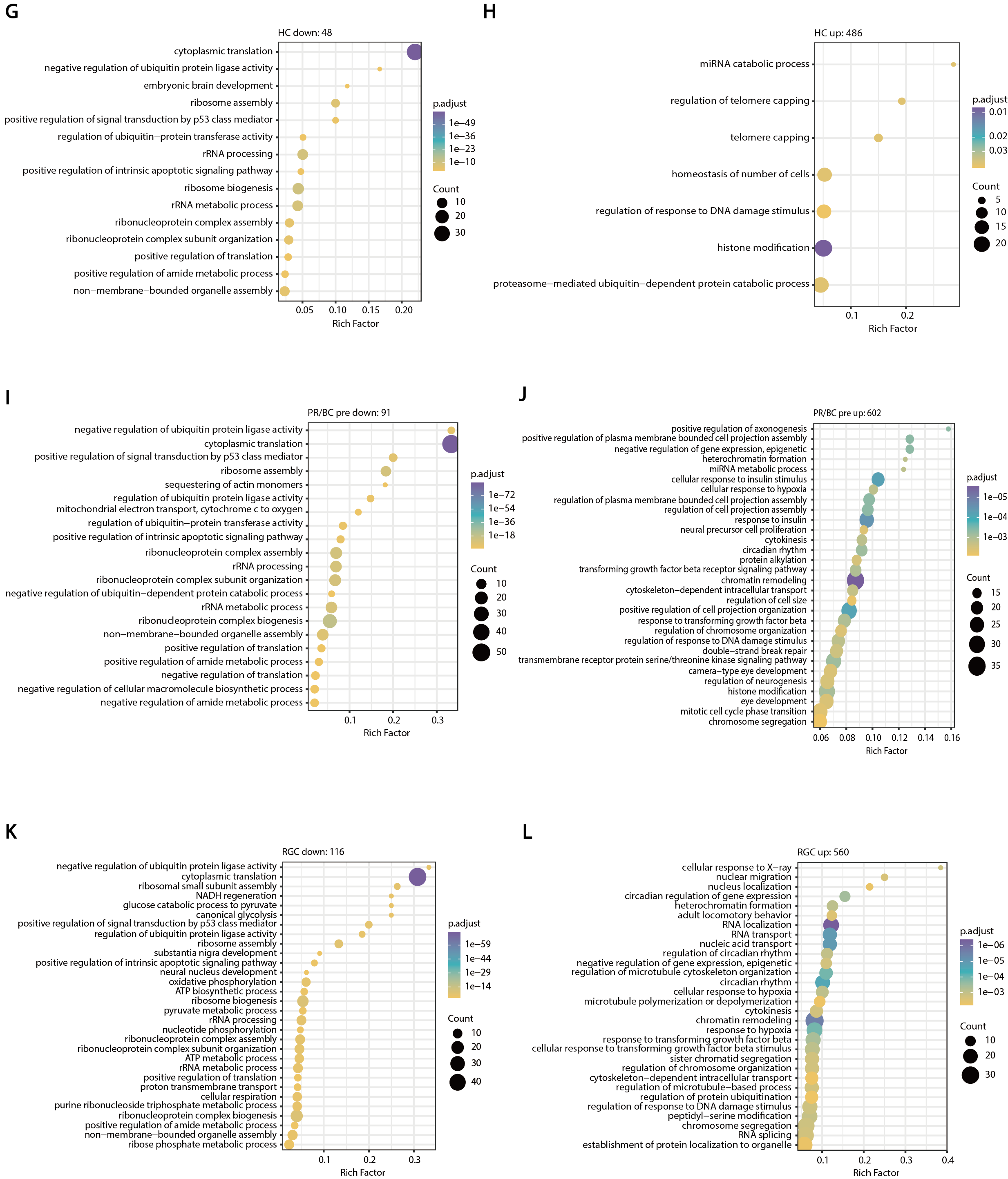


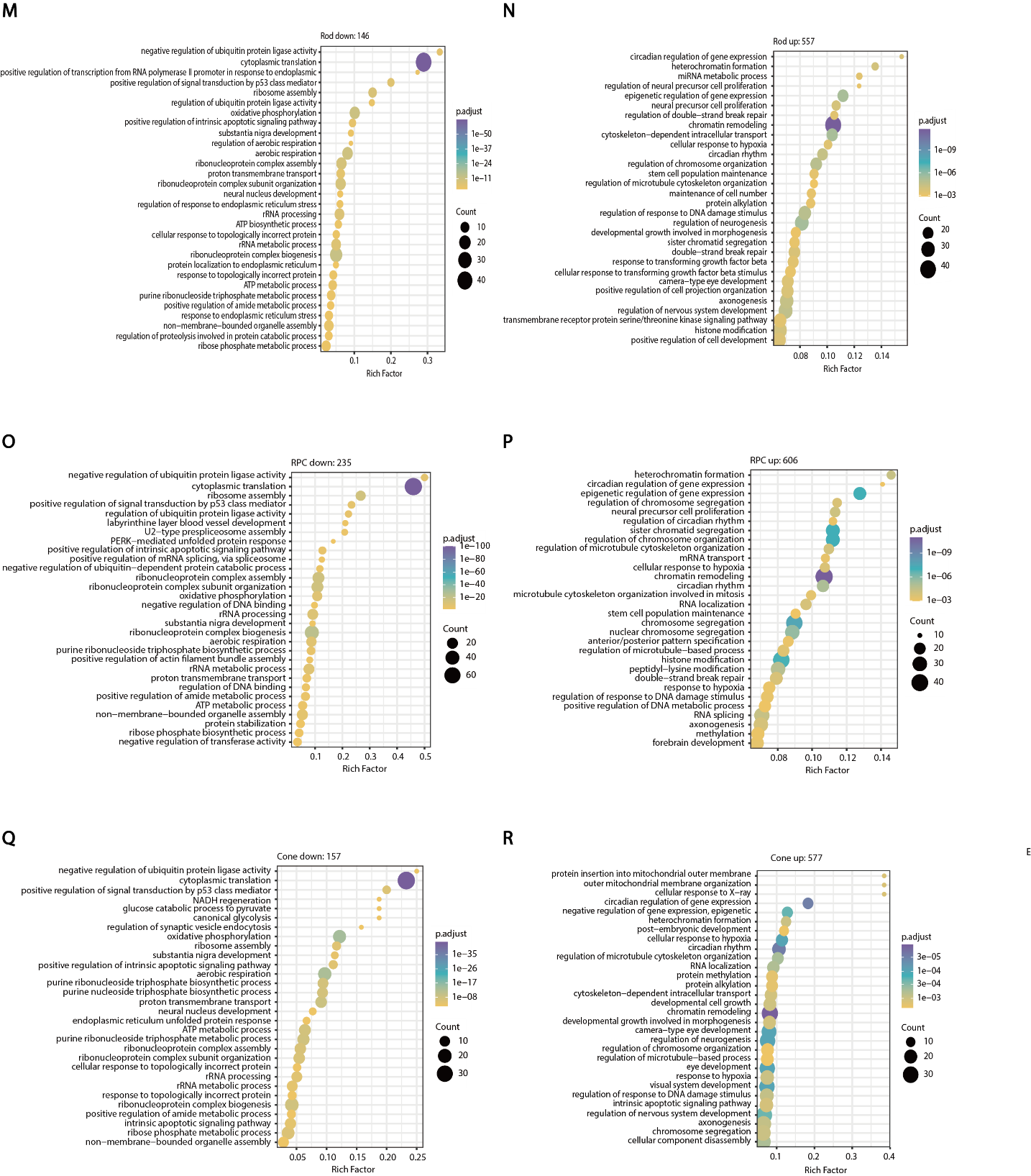


**Supplementary Fig. 3 GO analysis of differential gene expression in different cells of W14 retinal organoids after transfection with lentiviral vectors.** **A-B** GO analysis of 110 genes downregulated and 651 genes upregulated in amacrine cells after infection with lentiviral vectors. **C-D** GO analysis of 64 genes downregulated and 741 genes upregulated in bipolar cells after infection with lentiviral vectors. **E-F** GO analysis of 64 genes down-regulated and 689 genes up-regulated in horizontal/amacrine precursor cells after infection with lentiviral vectors. **G-H** GO analysis of 48 genes down-regulated and 486 genes up-regulated in horizontal progenitor cells after infection with lentiviral vectors. **I-J** GO analysis of 91 genes down-regulated and 602 genes up-regulated in photoreceptor/bipolar cell precursor cells after infection with lentiviral vectors. **K**-**L** GO analysis of 116 genes down-regulated and 560 genes up-regulated in retinal ganglion cells after infection with lentiviral vectors. **M-N** GO analysis of 146 genes down-regulated and 557 genes up-regulated in retinal rod cells after infection with lentiviral vectors. **O-P** GO analysis of 235 genes down-regulated and 606 genes up-regulated in retinal progenitor cells after infection with lentiviral vectors. **Q-R** GO analysis of 157 genes down-regulated and 557 genes up-regulated in cone cells after infection with lentiviral vectors.


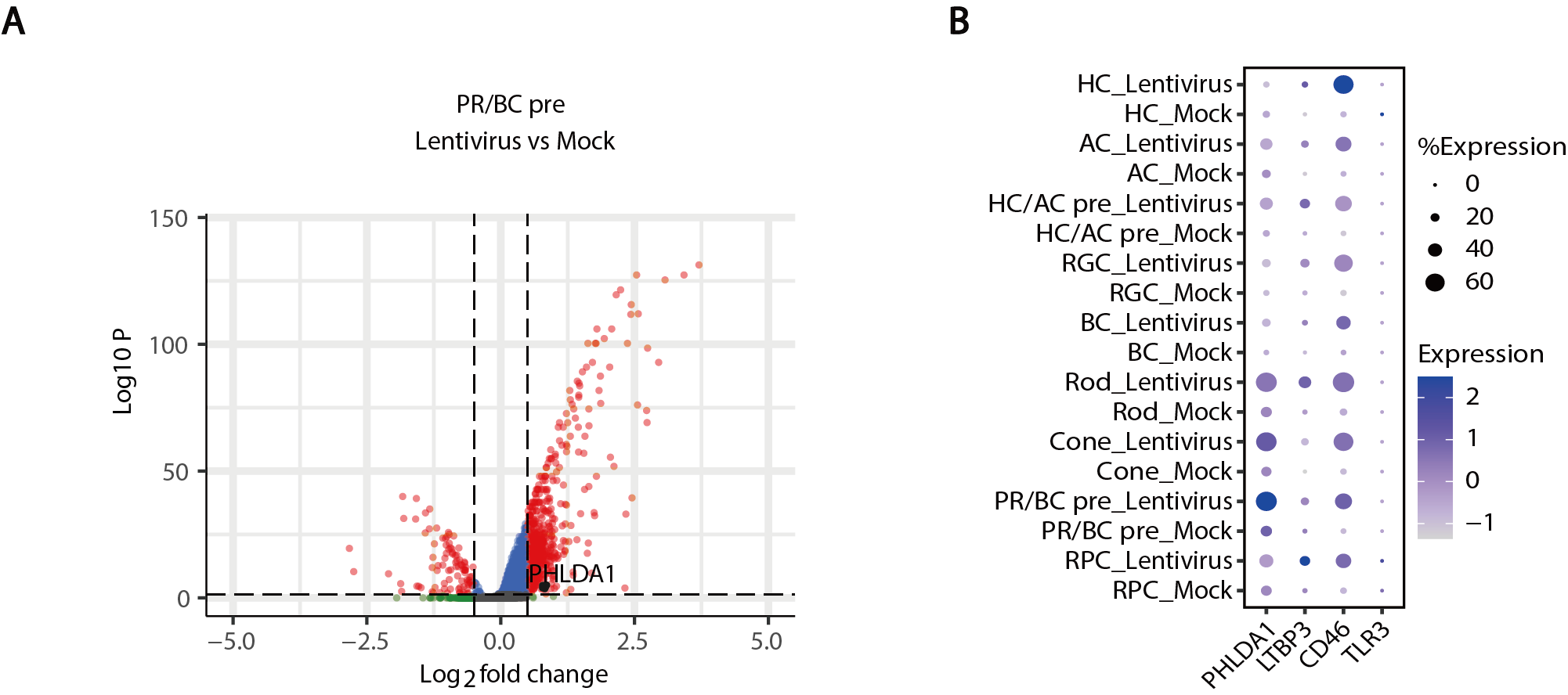


**Supplementary Fig. 4** **Infection of W14 retinal organoids with lentiviral vector leads to upregulation of *PHLDA1*.** **A** Volcano plot of differential gene distribution of photoreceptor /bipolar precursor cells after infection with lentivirus. **B** *PHLDA1* expression after lentivirus infection in different cells of retinal organoids.


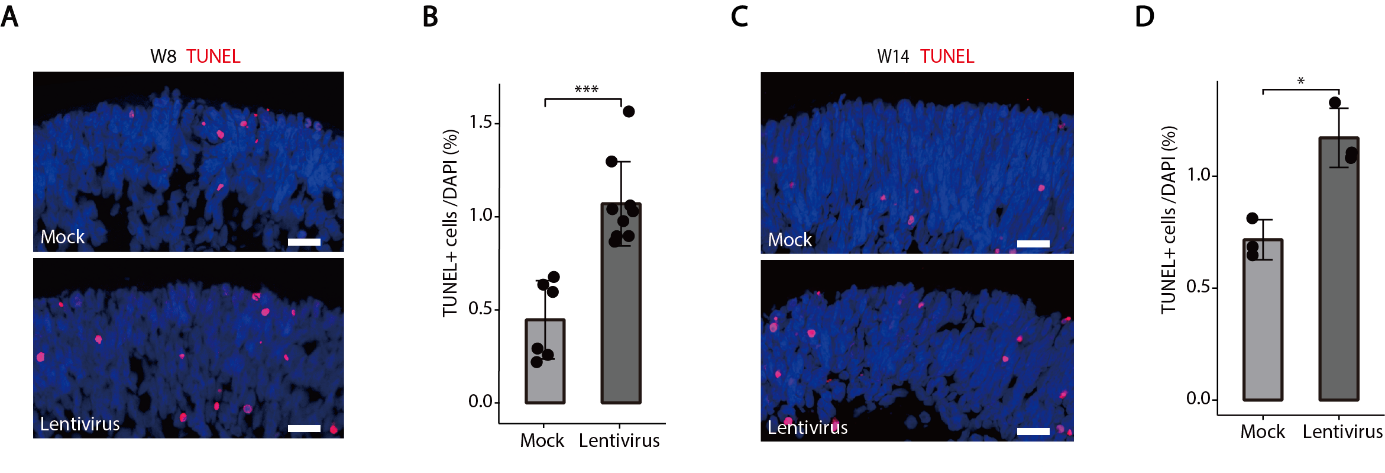


**Supplementary Fig. 5. Lentivirus infection of retinal organoids results in slight upregulation of apoptotic cells. A-B** Apoptosis cells were increased in W8 retinal organoids after infection with lentivirus. Scale bars, 20 μm. **C-D** Lentivirus vectors increased the number of apoptotic cells in W14 retinal organoids. Scale bars, 20 μm.


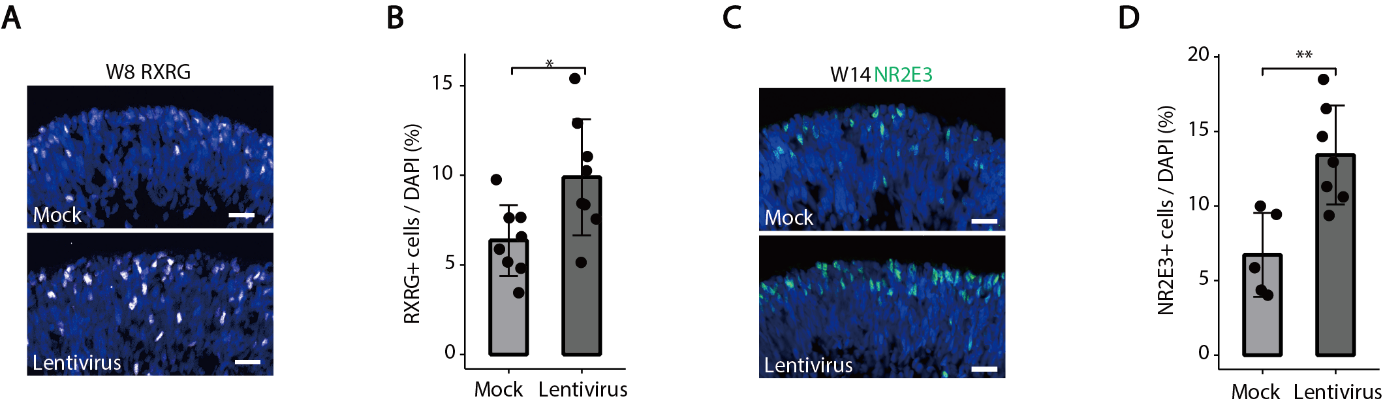


**Supplementary Fig. 6** **Infection of retinal organoids with lentiviral vectors leads to the increase of RXRG^+^ cells. A-B** The numbers of RXRG^+^ cone cells were significantly increased after infection of lentivirus. Scale bars, 20 μm. **C-D** The numbers of NR2E3^+^ rod cells were significantly increased after infection of lentivirus. Scale bars, 20 μm.


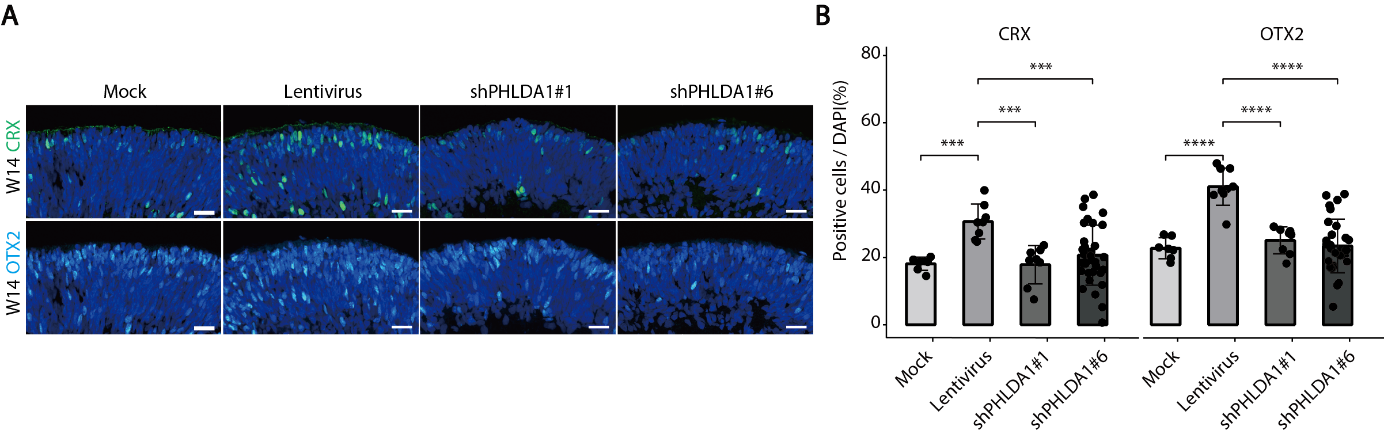


**Supplementary Fig. 7 Effects of PHLDA1 on photoreceptor differentiation. A-B** Immunofluorescence showed that knockdown of PHLDA1 in retinal organoids reduced the proportion of lentiviral-induced CRX^+^ and OTX2^+^ photoreceptor cells. Scale bars, 20 μm.
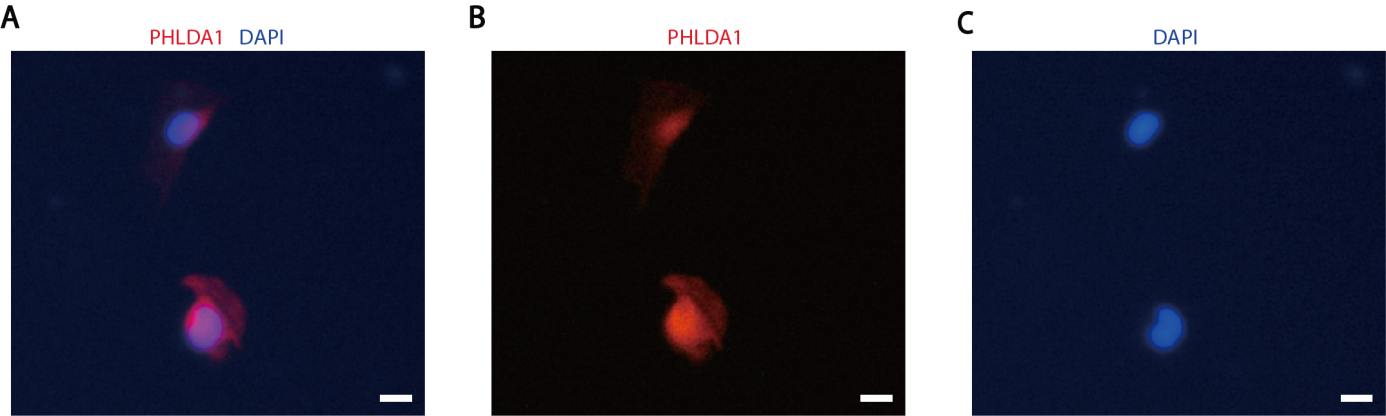


**Supplementary Fig. 8 PHLDA1 is distributed in the nucleus of Mesenchymal progenitor cells (MPCs). A-C** Subcellular localization of PHLDA1 in hESC differentiated MPCs (P6). Scale bars, 20 μm.


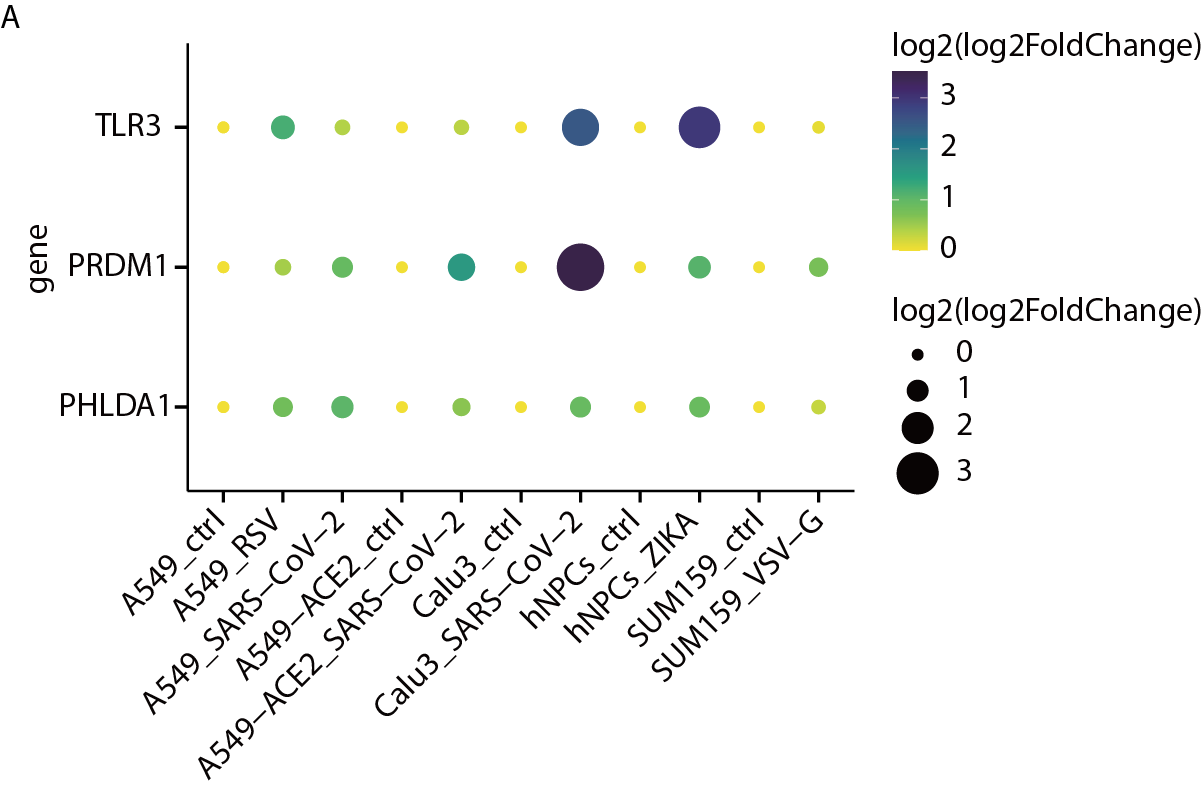


**Supplementary Fig. 9 Differential changes of PRDM1 in transcription levels after infection with different viruses. A** Transcriptome sequencing showed that compared with the mock group, transfection with SARS-Cov-2, RSV, ZIKA, and VSV-G viruses resulted in significantly up-regulated expression of PHLDA1 and PRDM1 mRNA.


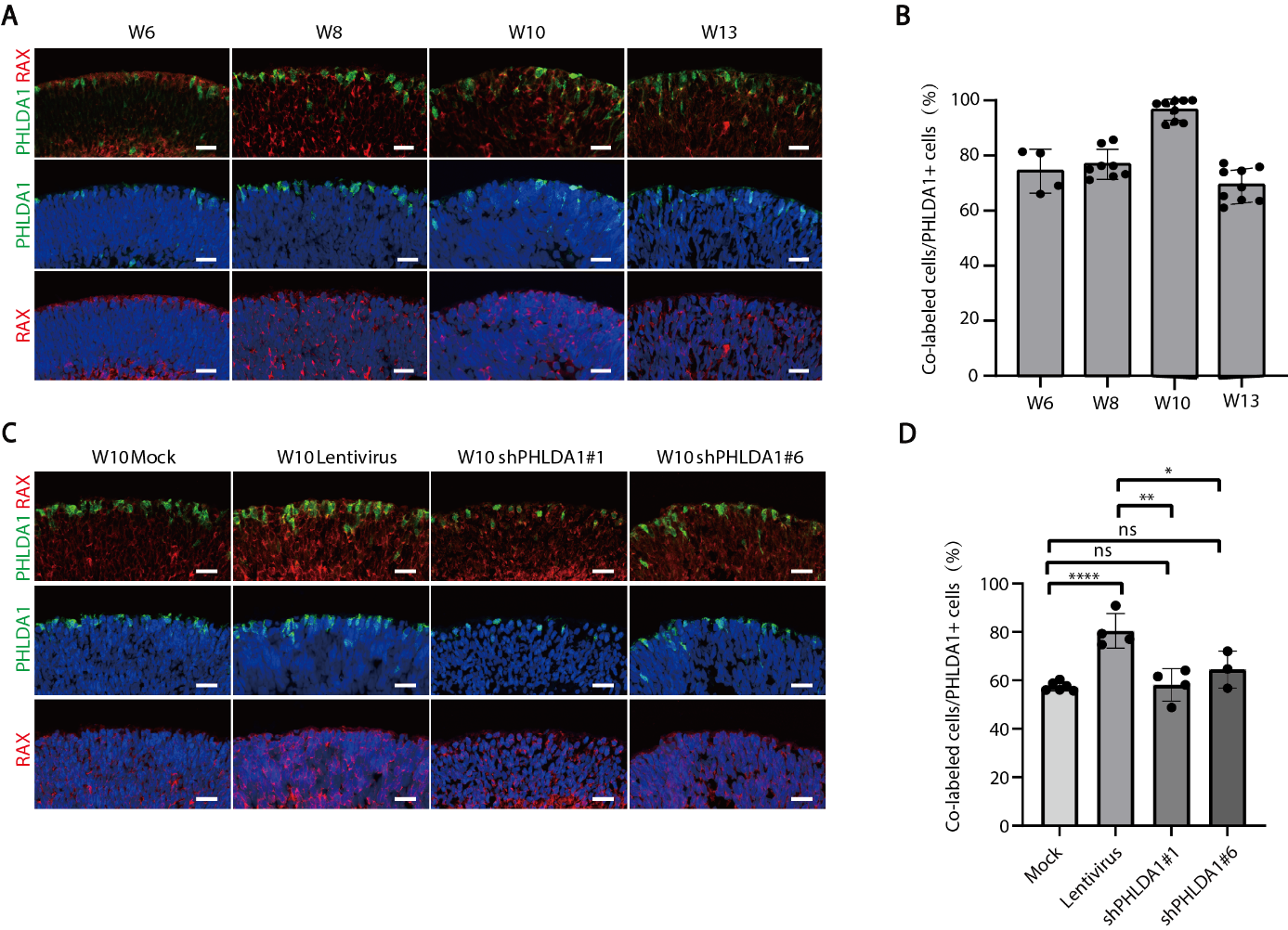


**Supplementary Fig. 10 PHLDA1 regulate the expression of RAX. A-B** Immunofluorescence co-localization staining results show that PHLDA1 is predominantly co-localized with RAX in retinal organoids at different developmental stages. Scale bars, 20 μm. **C-D** Lentivirus-induced RAX^+^ cell number in organoids was reversed by shRNA-mediated PHLDA1 knockdown (KD) compared to the mock control. Scale bars, 20 μm.


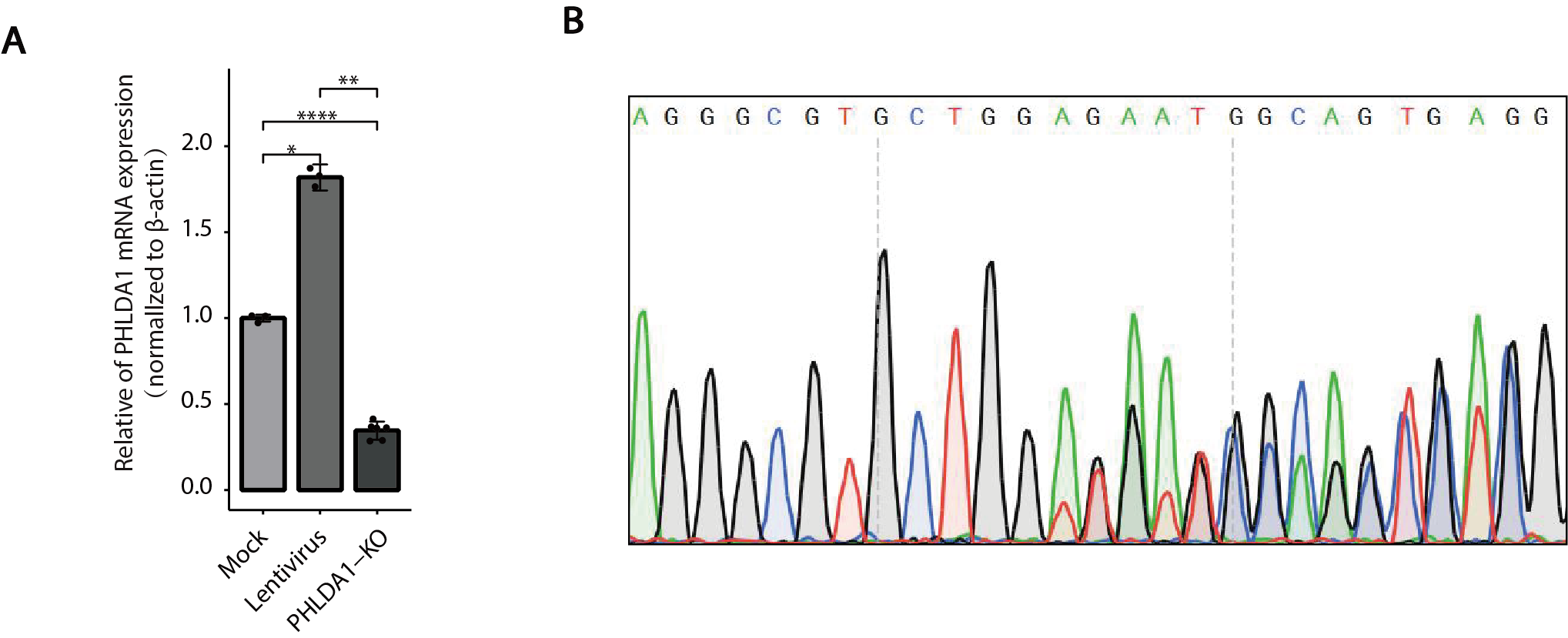


**Supplementary Fig. 11 Transcript levels and Sanger sequencing of *PHLDA1* in *PHLDA1* knockout Y79 cell line. A** *PHLDA1* mRNA expression in *PHLDA1*-KO Y79 cell line. **B** Sanger sequencing of Y79 cells confirms knockout of *PHLDA1*.

Appendix Table-1 shRNA target sequence

| **shRNA Name** | **Target Sequence** |
| --- | --- |
| **Scramble (lentivirus)** | **CCTAAGGTTAAGTCGCCCTCG** |
| **ShPHLDA1#1** | CGAGCACATTTCTATTGTCTT |
| **ShPHLDA1#6** | CAGATCAAGTAGTTTGGACAT |

Appendix Table-2 Immunofluorescence staining antibody information

| **Antibody** | **Dilution concentration** | Company | Catalog number |
| --- | --- | --- | --- |
| **PRDM1** | **1:100** | **Santa cruz** | **Cat# sc-47732** |
| **PHLDA1** | **1:100** | **Santa cruz** | **Cat# sc-23866** |
| **β-actin** | **1:1000** | **CST** | **Cat# 13E5** |
| **CRX** | **1:200** | **R&D** | **Cat# AF7085-SP** |
| **OTX2** | **1:50** | **Santa cruz** | **Cat# sc-514195** |
| **MKI67** | **1:200** | **Abcam** | **Cat# ab15580-100ug** |
| **Donkey anti-mouse 488** | **1:500** | **Abcam** | **Cat# ab50109** |
| **Donkey anti-rat 594** | **1:500** | **Abcam** | **Cat# ab150156** |
| **Donkey anti-mouse 594** | **1:500** | **Abcam** | **Cat# ab150112** |
| **Donkey anti-rabbit 647** | **1:500** | **Abcam** | **Cat# ab150063** |

Appendix Table-3 RT-qPCR primer sequence

| Primer Name | Sequence (5'-3') |
| --- | --- |
| **PHLDA1-qRT-F** | **CTTCACTGTGGTGATGGCAGAG** |
| **PHLDA1-qRT-R** | **CCTGACGATTCTTGTACTGCACC** |
| **PRDM1-qRT-F** | **CAGTTCCTAAGAACGCCAACAGG** |
| **PRDM1-qRT-R** | **GTGCTGGATTCACATAGCGCATC** |
| **ACTB-qRT-F** | **CACCATTGGCAATGAGCGGTTC** |
| **ACTB-qRT-R** | **AGGTCTTTGCGGATGTCCACGT** |
